# Carry-over effects in *Culex* species along a land use gradient with differences in microclimatic conditions

**DOI:** 10.1186/s13071-025-06903-y

**Published:** 2025-07-04

**Authors:** Carmen Villacañas de Castro, Johann Musculus, Esther Timmermann, Renke Lühken, Ellen Kiel, Felix Gregor Sauer

**Affiliations:** 1 Institute of Biology and Environmental Sciences, Carl Von Ossietzky Universität, Oldenburg, Germany; 2 Arbovirology and Entomology, Arbovirus Ecology, Bernhard-Nocht-Institute for Tropical Medicine, Hamburg, Germany

**Keywords:** Culicidae, Fitness, Carry-over effects, Land use, Microclimate

## Abstract

**Background:**

*Culex pipiens* sensu stricto (s.s.) and *Culex torrentium* are the major vectors of Sindbis, Usutu and West Nile virus in Europe. Both mosquito species typically breed in small artificial water containers (e.g. flower pots or rain barrels) and are the predominant mosquitoes in urbanised areas. The larval breeding conditions (e.g. temperature) can lead to carry-over effects on the emerging adults, which can influence their fitness traits (e.g. longevity or fecundity) and, finally, the vector capacity of a mosquito population. Our study aimed to investigate how the microclimatic heterogeneity across an urban area affects juvenile development and survival, as well as wing size, wing asymmetry and adult survival under heat stress of emerging adult *Cx. pipiens* s.s./*Cx. torrentium*.

**Methods:**

Experiments were conducted in Oldenburg (Lower Saxony, Germany) between 2021 and 2022. In a semi-field study, 45 artificial breeding habitats with 30 *Cx. pipiens* s.s./*Cx. torrentium* larvae were installed along a land use gradient, from vegetation-dominated to urban areas. The wings of all emerged mosquitoes were removed to measure wing size and wing asymmetry. Additionally, we tested the survival time of field-emerged adults exposed to 31 °C in the laboratory.

**Results:**

A piecewise structural equation model (SEM) was employed to simultaneously estimate the linear regression coefficients for various predicted relationships. The findings from the bivariate results align with the theoretical model derived from the SEM analysis. As sites with higher urbanisation indices had higher mean temperatures, mosquito development also differed along this urbanisation gradient. Results indicate that mosquitoes developing in warmer sites had shorter developmental times, and the highest juvenile survival occurred between 20 and 21 °C. Higher mean temperatures lead to lower adult survival times under heat stress and smaller wing centroid sizes. Finally, we showed that individuals with larger wing centroid sizes had lower asymmetries, which in turn also increased at higher maximum temperatures in the breeding sites, possibly indicating environmental stress.

**Conclusions:**

Our findings highlight the importance of microclimatic variation across urbanised areas on the development and fitness traits of *Culex pipiens* s.s./*Cx. torrentium* mosquitoes, emphasising the need to incorporate fine-scale microclimatic data into risk assessment models.

**Graphical abstract:**

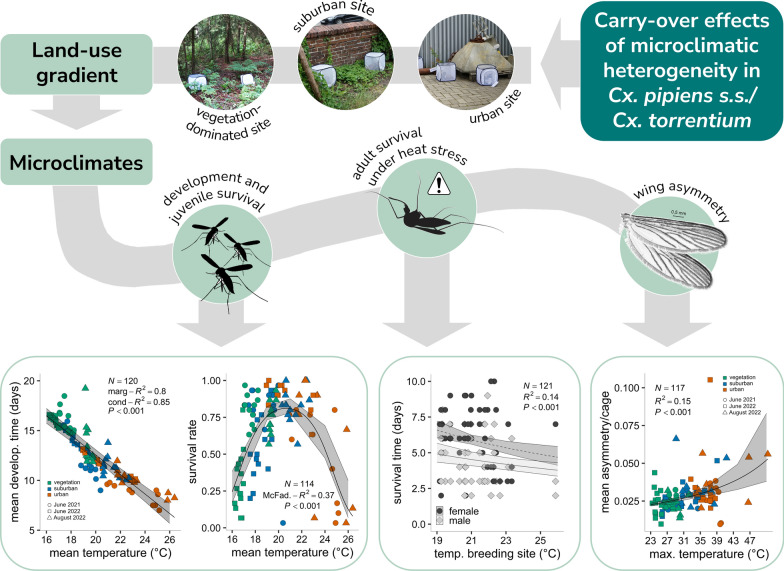

**Supplementary Information:**

The online version contains supplementary material available at 10.1186/s13071-025-06903-y.

## Background

One of the main concerns in global public health is vector-borne diseases, accounting for over 17% of all infectious diseases [1]. Over the past decades, there has been also an increase in the incidence of mosquito-borne diseases in Europe [2]. For example, the Sindbis virus, an alphavirus endemic to Finland, has caused annual disease outbreaks in humans since 2002 [3]. The Usutu virus, a flavivirus, is responsible for significant mortality in bird populations in Germany after several outbreaks since 2011 [4] and occasionally infects humans and other mammals [5, 6]. Finally, West Nile virus is an emerging arbovirus that circulates in birds and can affect human and animal health, in particular horses, as dead-end hosts [7–10]. In Central Europe, these viruses are predominantly transmitted by *Culex pipiens* sensu stricto (s.s.) and *Cx. torrentium* mosquitoes, which are widespread and among the most abundant mosquito species in urban areas [11–13], having a high vector competence [14] and broad host-feeding patterns [15].

Previous research has shown that urban areas with high environmental heterogeneity exhibit significant variability in microclimatic factors (e.g. temperature, humidity, radiation and wind speed) and mosquito breeding site availability, resulting in heterogeneous patterns of mosquito abundance, distribution and pathogen transmission at fine spatial scales [16–22]. Using a modelling approach, Wimberly et al. [21] demonstrated that the different microclimates associated with the heterogenic land use in urban areas could strongly affect the vectorial capacity of *Aedes albopictus* mosquitoes for dengue transmission. Therefore, extrapolating meteorological data from weather stations and macroclimate grids can lead to biased estimates when assessing the local risk for mosquito-borne pathogen transmission [23, 24]. This bias might be particularly relevant for the juvenile stages of container-breeding mosquitoes, such as *Cx. pipiens* s.s./*Cx. torrentium*. These are commonly found in anthropogenic breeding sites including ditches, small ponds, rain barrels or flower pots [25–27]. Unlike the mobile adults, juveniles cannot avoid unfavourable microclimatic conditions in small artificial breeding sites.

Temperature is a key factor influencing larval survival and development time [28]. Moreover, the environmental conditions during larval development can have variable effects on adult mosquito fitness – so-called carry-over effects. Ciota et al. [28] demonstrated in a laboratory study that different breeding site temperatures affect adult size, longevity, blood feeding and fecundity of *Culex* mosquitoes. Murdock et al. [20] showed that the microclimate in *Ae. albopictus* larval habitats varied among rural, suburban and urban sites, the latter being generally warmer and less humid. This led to decreased larval survival, smaller adult body sizes and lower per capita growth rates of mosquitoes in urban environments. Evans et al. [29] showed that the vector capacity of a mosquito population for the transmission of dengue virus by *Ae. albopictus* can be significantly misestimated if carry-over effects are not considered. Similarly, Brass et al. [30] integrated mosquito longevity into their stage-structured model, highlighting the importance of mosquito fitness traits to improve the accuracy of pathogen transmission risk predictions. The mentioned study used wing length as a proxy for mosquito fitness, as it is easily measured and correlated to longevity and fecundity [31]. Besides largely affecting wing length, increasing temperatures during larval development can also cause fluctuating asymmetry – minor deviations from perfect symmetry between the left and right wings [32] – in mosquitoes [33], a phenomenon that has also been observed in other dipteran insects [34–36]. This asymmetry is considered an indicator of environmental stress during larval development [37]. An understanding of the temperature-dependent breeding ecology and how the larval environment affects the fitness of the emerging adults is crucial to accurately predict mosquito-borne disease outbreaks [30]. However, most studies analysing carry-over effects in mosquitoes have focused on *Aedes* or *Anopheles* mosquitoes [29, 38–41], leaving significant knowledge gaps regarding the specific responses of *Cx. pipiens* s.s./*Cx. torrentium.*

This study explored the impact of microclimatic variations in urbanised areas on the development and fitness of *Culex* mosquitoes. We hypothesised that urbanisation influences the microclimate of mosquito breeding sites, which subsequently affects juvenile survival and development, as well as adult traits including wing size, wing asymmetry and survival under heat stress. Field and laboratory experiments were conducted between June 2021 and August 2022, examining the effects of breeding site temperatures on larval development and the carry-over effects in adult mosquitoes. To expose the larvae to different microclimates, the study focused on three land use classes representing varying urbanisation levels: low urbanisation (vegetation-dominated), intermediate urbanisation (suburban) and high urbanisation (urban).

## Methods

### Selection of the study sites and experimental setup

High resolution (10 m × 10 m) raster data on tree cover density (TCD) and imperviousness density (IMD) from the Copernicus Land Monitoring Service database (CORINE Land Cover 2018 v20) were used to identify suitable areas for the field study in Oldenburg (Lower Saxony, Germany; see Table 1). For each potential study site, the urbanisation index (UI) was calculated for a 20-m radius around the location by combining TCD and IMD as described by Gómez et al. [42]. Areas without TDC or IMD (e.g. cropland and grassland) are not represented by this UI and were not considered suitable in this study. The UI values were taken to designate land use classes: vegetation-dominated (0 ≤ UI ≤ 20), suburban (40 ≤ UI ≤ 60) and urban (80 ≤ UI ≤ 100). Values in between were not considered to obtain a clearer differentiation between the three UI classes. After the evaluation process was completed, three sites per land use class were selected for the latter field study (Table 1).

**Table 1:** List of selected study sites, their urbanisation index (UI) and land use class. The coordinates indicate the location where the reference logger was located at each site

Site code	UI	Land use class	Longitude	Latitude
GDH	0–20	Vegetation-dominated	53.1613714	8.15794772
STA	0–20	Vegetation-dominated	53.1428869	8.26516327
WOL	0–20	Vegetation-dominated	53.1577071	8.13562448
BGP	40–60	Suburban	53.1478366	8.19802953
GEF	40–60	Suburban	53.1498257	8.21607844
LUF	40–60	Suburban	53.1480571	8.18025408
AOF	80–100	Urban	53.1300539	8.22386332
BHL	80–100	Urban	53.1379640	8.26684610
WEW	80–100	Urban	53.1533789	8.16246891

To produce larvae for our 2-year field study, five artificial breeding sites were created using 10-L buckets filled with a mixture of water and hay infusion to attract gravid females, as according to Lampman and Novak [43]. The hay infusion was prepared by mixing 200 g of fresh hay (bunny NATURE FreshGrass Hay, Bunny Tierernährung, Melle, Germany) with 20 L of tap water and allowing it to infuse for a week with aeration every 48 h. Subsequently, this infusion was diluted in a 3:7 ratio with tap water. The artificial breeding sites were set up on the grounds of the Carl von Ossietzky University (locations: 53.146, 8.180; 53.147, 8.178) and inspected after 24 h for egg rafts, which were then collected and taken to the laboratory. Egg rafts were individually stored in trays with 500 mL of the same dilution used for the artificial breeding sites. In the first year, 10 egg rafts were collected and kept under controlled conditions in a climate chamber (KMF240-230 V, BINDER, Tuttlingen, Germany) at 24 °C ± 1 °C, 16:8 light–dark cycle and 80% ± 2% relative humidity. In the second year, 30 egg rafts were obtained in June and over 100 egg rafts in August and kept at room temperature, owing to unavailability of the climate chamber. Approximately 48 h after egg hatching, larvae from distinct egg rafts were combined and randomly selected to mitigate the effects of potential genetic differences between individual egg rafts. Subsequently, 30 larvae were transferred to 50-mL plastic bottles containing 20 mL of the breeding dilution for transportation to the study sites.

At each site, the setup consisted of five insect cages (AERARIUM, 30 cm × 30 cm × 30 cm, Bioform, Nürnberg, Germany) exposed at ground level with one breeding container inside each (45 cages in total). As breeding containers, we used polypropylene buckets for hydroculture (LENI CORONA 13/12, Lenz, Bergneustadt, Germany). At 3 cm below the upper edge, a hole was drilled to prevent overflow due to heavy rain events, protected with gauze to avoid any loss of larvae. The breeding containers were filled with 1 L of hay infusion (prepared as previously described) and equipped with a temperature logger inside (HOBO Pendant logger UA-001-64, Oncaset Computer Corporation, Bourne, MA, USA), which recorded the temperature hourly within the breeding site. Additionally, a masonite tile (20 cm × 3 cm × 0.4 cm) was placed vertically at an angle against the inner wall of the breeding container. This positioning provided a dry, stable surface that newly emerged adults could cling to as they expanded and dried their wings, thereby reducing the risk of drowning or damage.

### Field and laboratory studies

In total, 30 first-instar larvae of *Cx. pipiens* s.s./*Cx. torrentium* were added to each breeding container. These were checked daily, and if the breeding dilution content dropped below 0.8 L owing to evaporation, they were refilled with tap water to 1 L. After emergence, adults were collected from the cages using an aspirator, similar to the example developed by Vazquez-Prokopec et al. [44]. It was composed of a battery-powered blower (SEAFLO In-Line Blower SFIB1-270-02, Xiamen Doofar Outdoor, Xiamen, China), a pipe (Ø 10.5 cm × 22.5 cm) and catch bag (18 cm × 18 cm; Biogents, Regensburg, Germany) to collect adult mosquitoes. This field experiment was carried out in three time periods: June 2021, June 2022 and August 2022.

In addition, a laboratory assay was performed in August 2022 to analyse developmental time and juvenile survival rate as well as wing size and asymmetry under a controlled microclimate. This laboratory experiment consisted of 20 of the breeding containers as used in the field study with 30 larvae each, inside a climate chamber (same model as described previously) at a constant air temperature of 19 °C, 75% relative humidity and a 16:8 light–dark cycle.

### Carry-over effects on adult survival time under heat stress

In August 2022, four emerging adults (two female and two male) were randomly selected from each cage and taken directly to the lab to test their survival time under heat stress. They were kept individually in transparent 60 mL vials (Polyethylene, Ø 39 mm × 65 mm, Kartell Spa, Milano, Italy). The lid was modified with a gauze (pieces from cotton Esmarch bandages (DIN 13168), Carl Auffarth, Butjadingen, Germany) that allowed gas exchange. Adults were stored in a climate chamber at a constant air temperature of 31 °C with 70% ± 10% relative humidity and fed daily with 1 mL of 10% fructose solution on a cotton pad. The survival was recorded daily until their death and the number of survival days after emergence was documented.

### Wing measurements

All emerged and collected adults were frozen at −15 °C. Subsequently, the wings were dissected under a Leica M205C Binocular Microscope (Leica Microsystems, Heerbrugg, Switzerland), mounted in the embedding medium Euparal (Carl Roth GmbH, Karlsruhe, Germany) on microscope slides (cut frosted, Thermo Scientific, Menzel-Gläser, Gerhard Menzel, Braunschweig, Germany) and coverslips (24 mm × 40 mm; Menzel-Gläser, Gerhard Menzel, Braunschweig, Germany). The mounted wings were photographed using a binocular (described above), attached to a Leica DMC4500 Digital Microscope camera with a 20× magnification.

Wings were measured using the image processing program ImageJ [45]. To measure wing size, geometric morphometric wing analysis was applied as described by Bookstein [46]. In the first step, 18 landmarks (see Additional file 1: Supplementary Fig. S1) on the anatomical junctions of the wing veins were selected in accordance with Wilke et al. [47]. The landmark coordinates of each pair of wings were digitalised by a single observer to avoid bias during landmark collection. The raw landmark coordinates were then used to calculate the centroid size for each wing, which is defined as ‘the square root of the sum of squared distances between the centroid and each landmark’ [46]. Centroid size can be used as a proxy for body size [48, 49]. In addition, the centroid size of the right and left wings of each mosquito was used to calculate the absolute difference, providing a quantitative measure of wing asymmetry.

### Statistical analysis

**Fig. 1:**
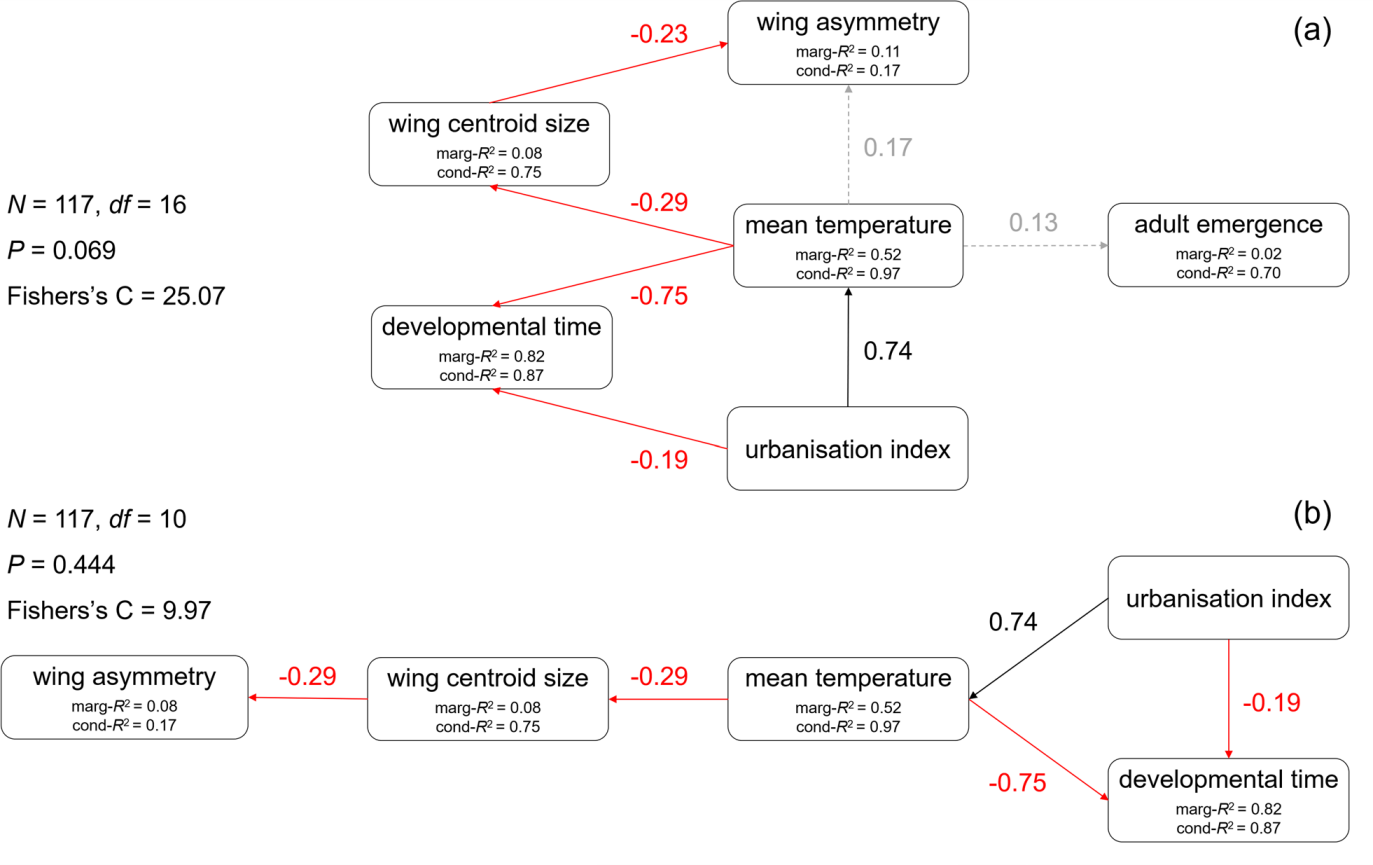
Piecewise structural equation models (*N* = 117 cages) exploring the direct and indirect effects of urbanisation index and mean temperature on different life-history traits. Diagram (**a**) shows the initial piecewise SEM, while diagram (**b**) shows the final optimised model. The boxes represent the different variables measured. Solid arrows represent significant (*P* < 0.05) unidirectional relationship between variables. Black represents positive and red represents negative relationships. Dashed and grey arrows represent non-significant relationships. We report the standardised coefficients next to the arrows. The amount of variation explained by the model is given as marginal and conditional *R*^2^ in the boxes of their response variables. Goodness-of-fit metrics are on the left of the diagrams

All statistical analyses were performed using R (Version 4.0.5) for statistical computing [50] and the interface RStudio [51]. In a first step, we performed a piecewise structural equation model (SEM) [52] where we verified the theoretical model outlined in our hypothesis, including the following variables: UI, mean temperature within the breeding sites (hereafter referred to as mean temperature), mean developmental time per cage, adult emergence per cage, mean wing centroid size per cage and mean wing asymmetry per cage, with field site and trial as crossed random terms. Mean wing asymmetry had a strong gamma distribution, not yet supported by these models. Therefore, this variable was log-transformed into a normal distribution, and the complete set of linear regressions were fitted simultaneously. Survival time under heat stress was not included in the model as only a subsample of the emerged adults was used for this trial, and the laboratory control data were not considered either. The SEM was implemented with the piecewiseSEM package [52]. The DiagrammeR package [53] was used to visualise the path diagrams modified to represent Fig. 1a, b.

Following the SEM modelling, bivariate analyses on the significant paths identified by SEM were applied as a confirmatory step, providing more detailed insights into the specific relationships. Linear mixed models (LMM) [54] were used to analyse the correlation between UI and mean temperatures, mean larval developmental time per cage and land use class (including the laboratory control), as well as mean larval developmental time and mean temperatures. The function lmer from package lme4 [55] was used, including trial and field site as crossed random terms.

To analyse the juvenile survival probability (measured as the proportion of emerging adults per cage from a maximum of 30 larvae) as a function of land use class, including the lab control, we used a generalised linear mixed model (GLMM) [56] with trial and field site as random terms, a binomial error distribution and logit link function, using function glmer from package lme4 [55]. Survival probability was analysed as a function of mean, maximum and minimum temperature and compared the *R*^2^ values, as Akaike information criterion (AIC) and Bayesian information criterion (BIC) values are not defined for quasibinomial models. Mean temperature was the best predictor for survival probability and was analysed using a polynomial generalised Linear Model (GLM) [57] with a binomial error distribution with logit link function and a correction for over-dispersed data.

Survival time of the adult mosquitoes under heat stress (31 °C) was analysed as a function of breeding site mean, maximum and minimum temperature and mean temperature was selected as the best predictor on the basis of a lower quasilikelihood information criterion (QIC) value. A generalized estimating equation (GEE) [58] model was used, with function geeglm from the package geepack [59–61], with a gamma error distribution and an inverse link function, correlation structure exchangeable and field site included as a random term.

Wing centroid size for each emerging adult was calculated by taking the mean between the left and right wings. The wing size of female and male specimens was analysed separately as both sexes are well known for differences in their body and wing size [62]. Individual female and male wing centroid size was analysed separately as a function of mean, maximum and minimum temperature; selection was done by comparing AIC and marginal *R*^2^ values. Mean temperature was selected as the best predictor and analysed using an LMM [54], with the function lmer from package lme4 [55] including cage as random term.

Mean wing asymmetry per cage (including the laboratory control) was analysed as a function of mean centroid size per cage. Moreover, despite being not significant in the SEM, mean wing asymmetry per cage was also analysed as a function of mean, maximum and minimum temperature, and the best predictor was selected on the basis of QIC, choosing the model with the lowest value. In this case, the best predictor was maximum temperature. In both cases, a GEE [58] model was used, function geeglm from the package geepack [59–61], with a gamma error distribution, an inverse link function, correlation structure exchangeable and field site included as a random term.

Additionally, the packages raster [63], sp [64, 65] and rgdal [66] were used to calculate the UI of the breeding sites. The calculation of the centroid sizes was carried out using the package geomorph [67, 68]. The package MuMIn [69] was used to obtain *R*^2^ values for the models. Finally, the ggplot2 package [70] was used to create figures, and the emmeans package [71] was used to calculate the confidence intervals represented in the bar graph. A Tukey-adjusted pairwise contrast test was also performed using the lsmeans package [72] to test for significant differences among land use classes for the mean developmental time.

## Results

Overall, the initial SEM model had a good fit of the covariance matrix, with a high *P*-value indicating that the model could not be rejected (*P* = 0.069, Fisher’s *C* value = 25.07, df = 16). However, the direct effects of mean temperature on adult emergence per cage and mean wing asymmetry per cage were not significant (Fig. 1a; Table 2). Therefore, we deleted the non-significant regressions, obtaining the final optimal model shown in Fig. 1b (*P* = 0.444, Fisher’s *C* value = 9.97, df = 10; Table 2). The standardised coefficients indicated a positive direct effect of UI on mean temperature (0.74), while a negative effect was observed for mean developmental time per cage (−0.19). Simultaneously, mean temperature had a negative direct effect on mean developmental time per cage (−0.75), as well as a negative direct effect on mean centroid size per cage (−0.29) and, therefore, an indirect negative effect on mean wing asymmetry per cage through mean centroid size per cage (−0.29). However, these two final pathways, despite being significant, had very low marginal coefficients of determination, *R*^2^ (Fig. 1b; Table 2). All other tests of directed separation (which test the missing links among the variables) were also not significant.

**Table 2:** Results from the piecewise structural equation modelling (SEM) showing the initial model (a) and the final optimal model (b)

(a) Initial model				
Response	Predictor	Stand. estimate	SE	*P*
Mean temperature	Urbanisation index	0.7420	0.0062	< 0.0001***
Adult emergence	Mean temperature	0.1248	0.4939	0.3879
Developmental time	Mean temperature	−0.7532	0.0890	< 0.0001***
Developmental time	Urbanisation index	−0.1903	0.0056	0.0183*
Wing centroid size	Mean temperature	−0.2935	0.0178	0.0009***
Wing asymmetry	Wing centroid size	−0.2304	0.0764	0.0249*
Wing asymmetry	Mean temperature	0.1672	0.0162	0.1069

(b) Final optimal model				
Response	Predictor	Stand. estimate	SE	*P*
Mean temperature	Urbanisation index	0.7420	0.0062	< 0.0001***
Developmental time	Mean temperature	−0.7532	0.0830	< 0.0001***
Developmental time	Urbanisation index	−0.1903	0.0056	0.0183*
Wing centroid size	Mean temperature	−0.2935	0.0178	0.0009***
Wing asymmetry	Wing centroid size	−0.2889	0.0739	0.0039**

Significance is shown with asterisks (*P < 0.05; **P < 0.01; ***P < 0.001)

**Fig. 2:**
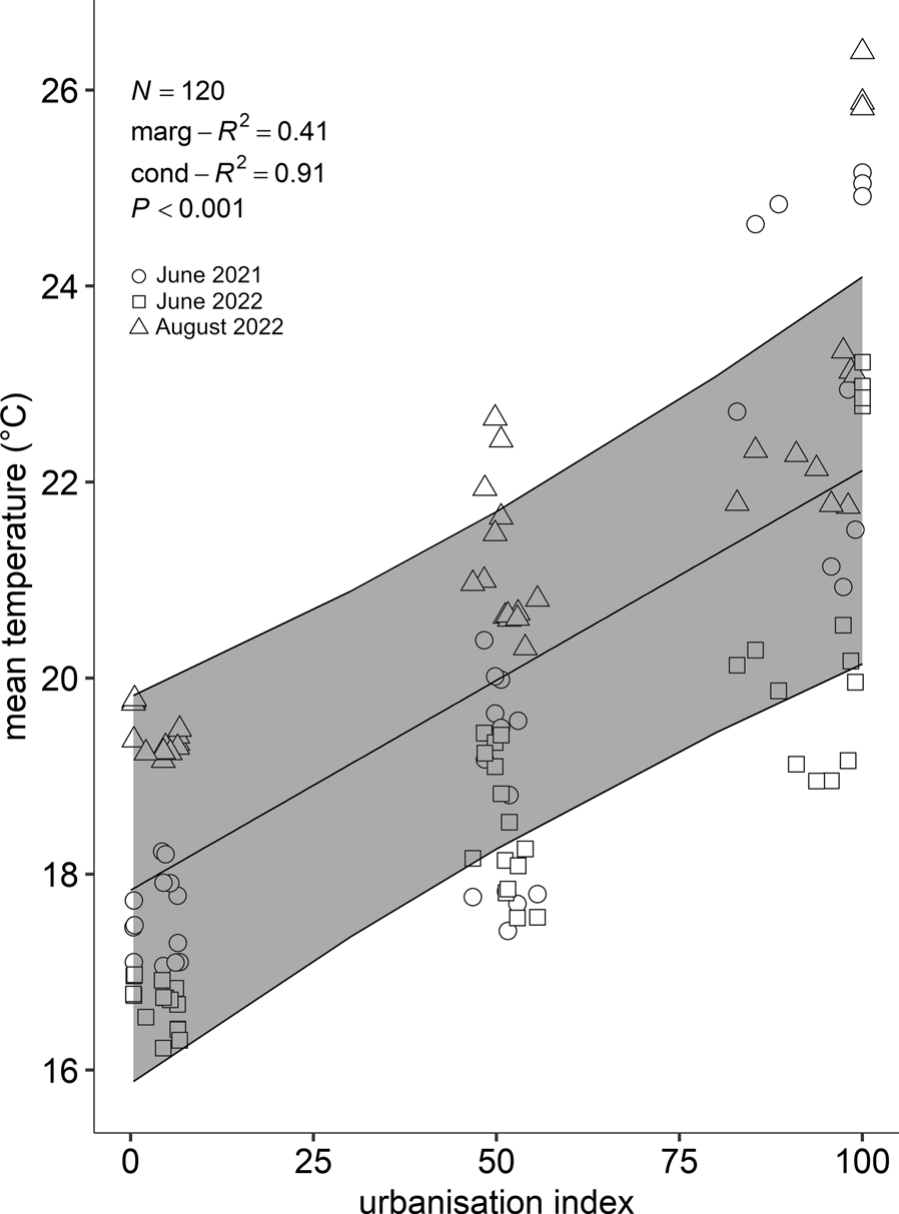
Mean temperature (°C) within the breeding dilution increases as a function of urbanisation index (UI classes: 0 ≤ UI ≤ 20 = vegetation-dominated; 40 ≤ UI ≤ 60 = suburban; 80 ≤ UI ≤ 100 = urban). The graph shows the data points for the three trials: black dots for June 2021, black squares for June 2022 and black triangles for August 2022. The model best-fitting line with 95% CI is represented (LMM, random terms = field site and trial)

The LMM confirmed a positive correlation between UI and mean temperatures. The mean temperature significantly increased for sites with higher UI (Fig. 2: *y* = 17.84 + 0.04*x*; chi-squared test, *χ*^2^ = 19.27, degrees of freedom (df) = 1, *P* < 0.0001, marginal *R*^*2*^ = 0.41, conditional *R*^*2*^ = 0.91) and were up to 4 °C warmer in urban areas compared with vegetation-dominated areas.

**Fig. 3:**
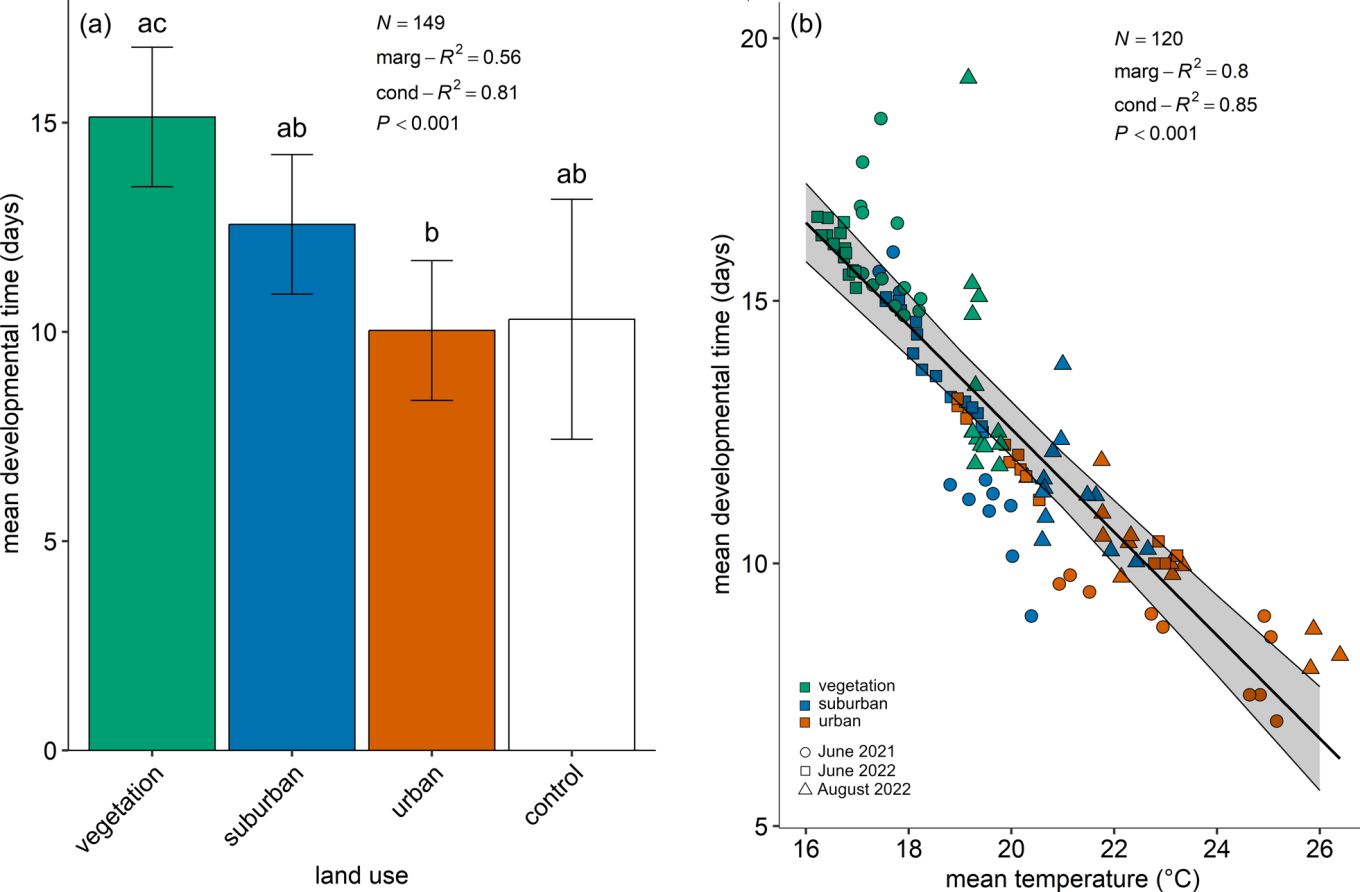
**a**, Mean larval developmental time per cage (in days) as a function of land use class, including the laboratory control. The graph shows the model estimates (± standard error, SE). Different lowercase letters indicate significant differences between treatments (LMM, random terms = field site and trial). **b**, Mean larval developmental time per cage (in days) as a function of mean temperature (°C) within the breeding dilution (*y* = 32.21 − 0.98*x*). The graph shows the data points for the different land use classes (classes: 0 ≤ UI ≤ 20 = vegetation-dominated; 40 ≤ UI ≤ 60 = suburban; 80 ≤ UI ≤ 100 = urban) and the three trials (dots for June 2021, squares for June 2022 and triangles for August 2022). The model best-fitting line with 95% CI is represented (LMM, random terms = field site and trial)

The mean larval developmental time per cage was analysed in relation to land use class and compared with the control group, revealing a statistically significant correlation (Fig. 3a: *χ*^2^ = 44.76, df = 3, *P* < 0.0001, marginal *R*^2^ = 0.56, conditional *R*^2^ = 0.81). After performing a pairwise contrast test with a Tukey adjustment (Table 3), it was shown that the mean larval developmental time of the laboratory control group (mean ± SE = 10.30 ± 1.45 days) did not significantly differ from the mean developmental time of larvae breeding in vegetation-dominated (15.13 ± 0.84 days), suburban (12.57 ± 0.84 days) and urban areas (10.03 ± 0.84 days). However, there were significant differences between larvae breeding in vegetation-dominated and urban areas (*P* = 0.003). This effect aligns with the observation that mean larval developmental time was significantly shorter as temperatures increased (Fig. 3b: *y* = 32.21 − 0.98*x*; *χ*^2^ = 192.06, df = 1, *P* < 0.0001, marginal *R*^*2*^ = 0.80, conditional *R*^*2*^ = 0.85).

**Table 3:** Pairwise contrast tests with a Tukey adjustment for the mean larval developmental time per cage (in days) as a function of land use class (including the laboratory control)

Land use	Vegetation	Suburban	Urban
Vegetation	–	–	–
Suburban	0.0611	–	–
Urban	0.0025**	0.0641	–
Control	0.1196	0.5760	0.9983

Significance is shown with asterisks (*P < 0.05; **P < 0.01; ***P < 0.001)

**Fig. 4:**
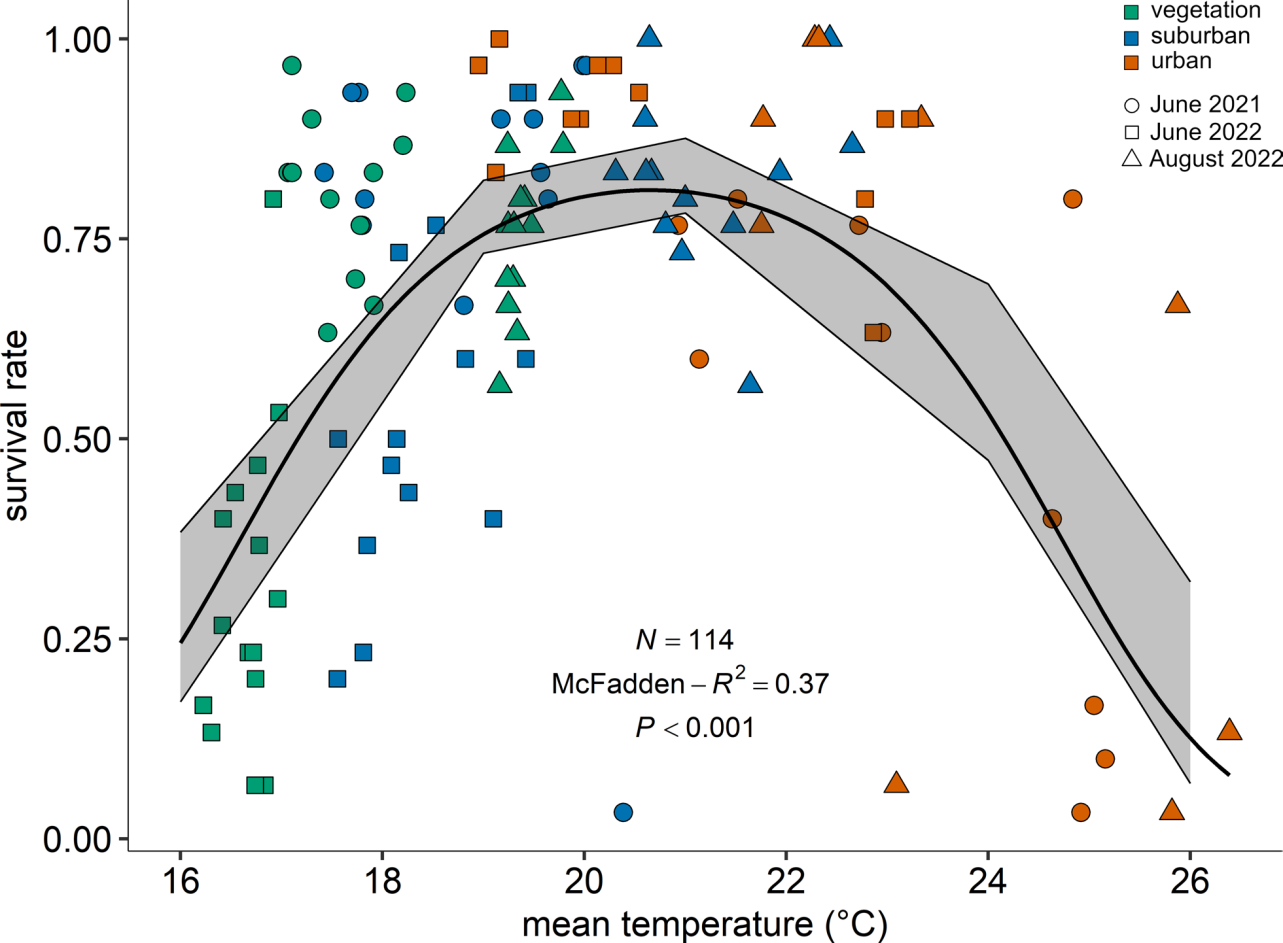
Mean survival rate per cage as a function of mean temperature (°C) within the breeding site (*y* = exp(4.92*x* − 0.12*x*^2^ − 49.33)/1 + exp(4.92*x* − 0.12*x*^2^ − 49.33)). The graph shows the data points for the different land use classes (classes: 0 ≤ UI ≤ 20 = vegetation-dominated; 40 ≤ UI ≤ 60 = suburban; 80 ≤ UI ≤ 100 = urban) and the three trials (dots for June 2021, squares for June 2022 and triangles for August 2022) for visualisation purposes. The model best-fitting line with 95% CI is represented (polynomial GLM Binomial with correction for over-dispersed data)

The juvenile survival probability (measured as the proportion of emerging adults per cage) was analysed as a function of land use class and compared with the control group. There were no significant differences in the survival probability of mosquitoes breeding in the lab (71.30% ± 4.75%), vegetation-dominated (62.70% ± 4.05%), suburban (70.40% ± 3.87%) and urban areas (65.50% ± 5.31%). Afterwards, we analysed the survival probability as a function of mean temperature, obtaining the optimal temperature curve shown in Fig. 4 (*y* = exp(4.92*x* − 0.12*x*^2^–49.33)/1 + exp (4.92*x* − 0.12*x*^2^ − 49.33); temperature: *F*-test, *F* = 65.33, df = 1, *P* < 0.0001; temperature^2^: *F* = 65.09, df = 1, *P* < 0.0001; McFadden *R*^2^ = 0.37). According to our model, individuals have the highest survival probability (approximately 80%) to the adult stage at a mean temperature of 20–21 °C. A survival probability above 50% is achieved at mean temperatures between 17 and 24 °C. Mean temperatures above or below that span rapidly reduce the survival probability.

**Fig. 5:**
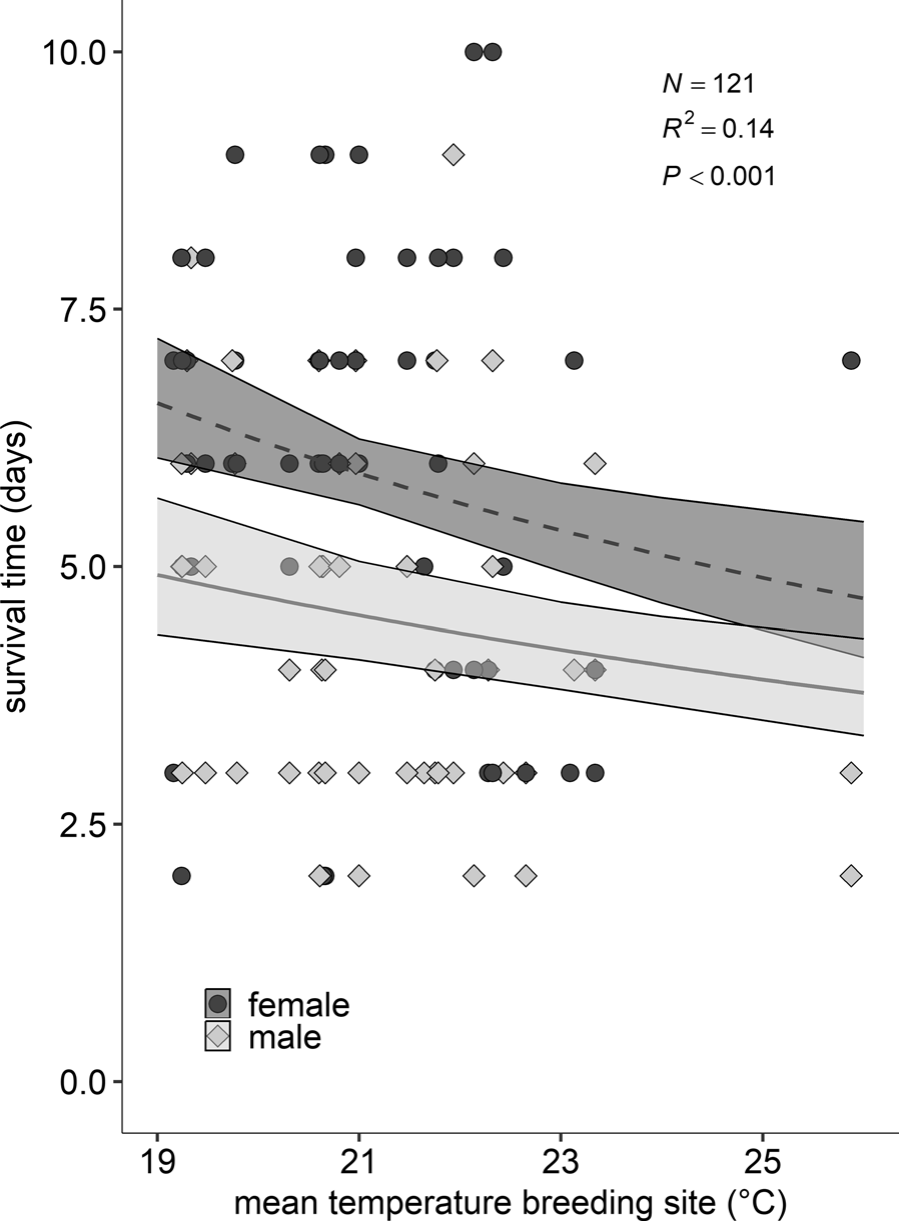
Survival time of adult mosquitoes (in days) under heat stress (constant 31 °C) as a function of mean temperatures in their breeding sites and sex. The graph shows the data points as dark dots for females and grey diamonds for males as well as the model best-fitting lines (*y*_f_ = 1 / (−0.02 + 0.0088*x*); *y*_m_ = 1 / (0.04 + 0.0088*x*)) and 95% CI (GEE gamma, random term: field site). *m:* males, *f:* females

A total of 71 female and 67 male specimens were included in the experiment to evaluate the survival time under heat stress (31 °C). In several breeding sites, no specimen or fewer than two females and two males emerged, preventing the inclusion of four specimens per breeding site. The survival time of the mosquitoes ranged from 2 to 10 days (Fig. 5). The mean temperature was the best predictor for the survival time under heat stress. Specifically, survival time significantly decreased with increasing mean temperatures (Fig. 5: *χ*^2^ = 10.50, df = 1, *P* = 0.001). There were significant differences in the survival time between the sexes (*χ*^2^ = 17.20, df = 1, *P* < 0.0001; *y*_females_ = 1 / (−0.02 + 0.0088*x*), *y*_males_ = 1 / (0.04 + 0.0088*x*); *R*^2^ = 0.14), with female mosquitoes living, on average, 1–2 days longer than males (Fig. 5). However, the interaction between both terms was not significant, i.e. the effect of temperature on adult survival time under heat stress was consistent across the sexes.

**Fig. 6:**
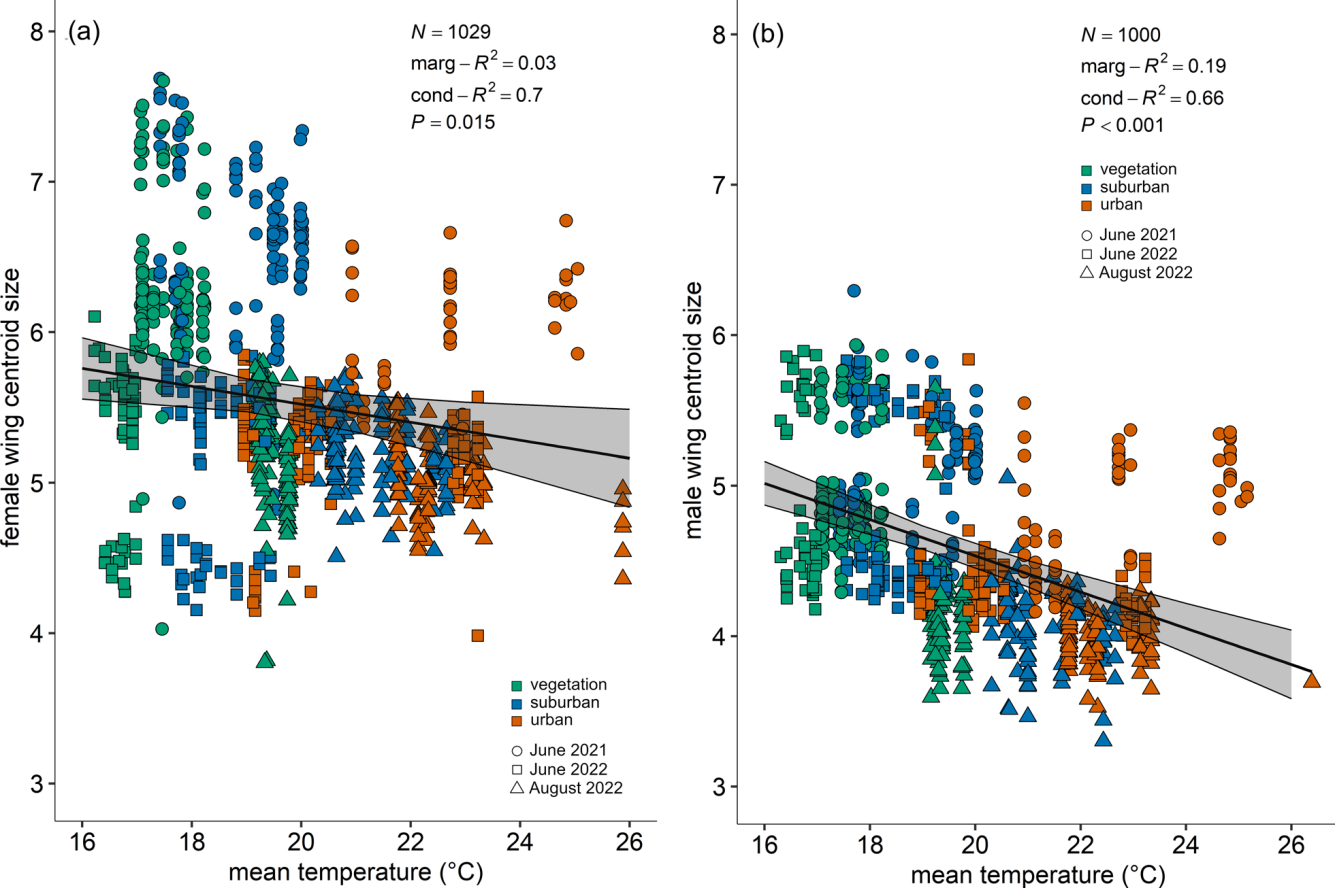
**a**, Female wing centroid size as a function of mean temperature (°C) within the breeding dilution (*y*_f_ = 6.71 − 0.06*x*). **b**, Male wing centroid size as a function of mean temperature (°C) within the breeding dilution (*y*_m_ = 6.94 − 0.12*x*). The graphs show the data points for the different land use classes (classes: 0 ≤ UI ≤ 20 = vegetation-dominated; 40 ≤ UI ≤ 60 = suburban; 80 ≤ UI ≤ 100 = urban) and the three trials (dots for June 2021, squares for June 2022 and triangles for August 2022). The models best-fitting lines with 95% CI are represented (LMM, random term: cage)

Overall, females had larger wing centroid sizes than male specimens. Both female (*χ*^2^ = 5.89, df = 1, *P* = 0.015, marginal *R*^2^ = 0.03, conditional *R*^2^ = 0.70; *y*_f_ = 6.71 − 0.06*x*) and male (*χ*^2^ = 48.47, df = 1, *P* < 0.0001, marginal *R*^2^ = 0.19, conditional *R*^2^ = 0.66; *y*_m_ = 6.94 − 0.12*x*) specimens exhibited a smaller wing centroid size with increasing mean temperatures (Fig. 6).

**Fig. 7:**
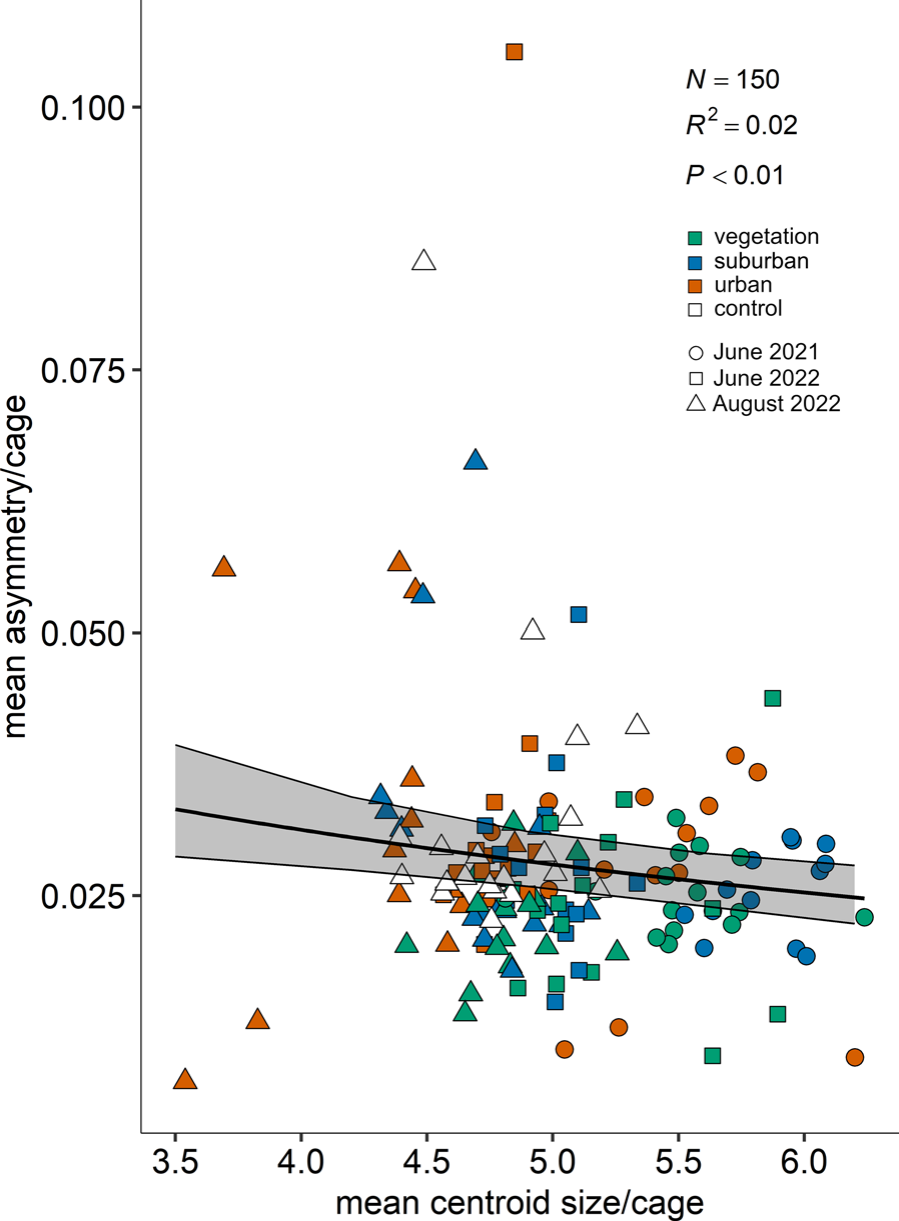
Mean wing asymmetry per cage as function of wing centroid size. The graphs show the data points for the different land use classes (classes: 0 ≤ UI ≤ 20 = vegetation-dominated; 40 ≤ UI ≤ 60 = suburban; 80 ≤ UI ≤ 100 = urban) and the three trials (dots for June 2021, squares for June 2022 and triangles for August 2022). The model best-fitting line with 95% CI is represented (*y* = 1 / (16.89 + 3.77*x*)) (GEE, random term: site)

**Fig. 8:**
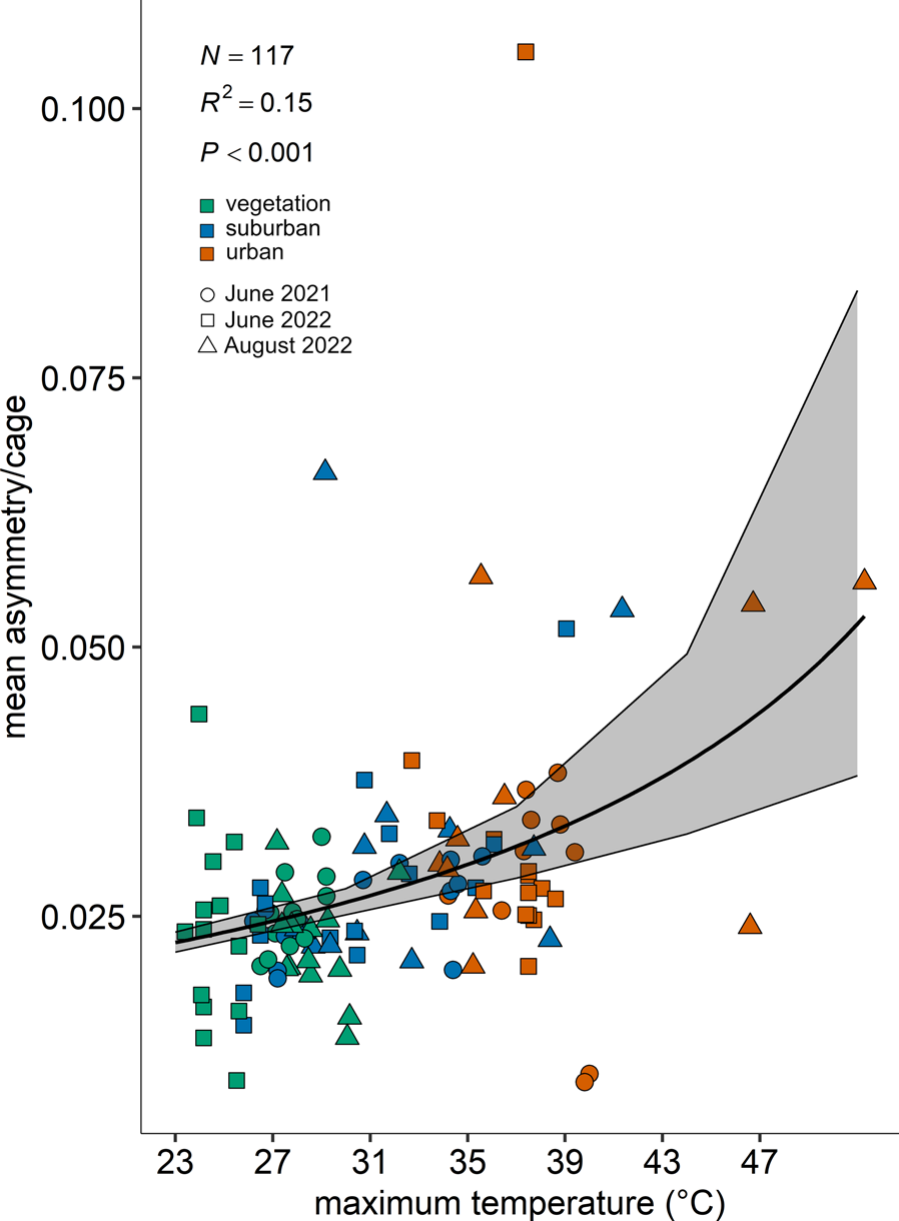
Mean wing asymmetry per cage as a function of maximum temperatures (°C) within the breeding dilution. The graphs show the data points for the different land use classes (classes: 0 ≤ UI ≤ 20 = vegetation-dominated; 40 ≤ UI ≤ 60 = suburban; 80 ≤ UI ≤ 100 = urban) and the three trials (dots for June 2021, squares for June 2022 and triangles for August 2022). The model best-fitting line with 95% CI is represented (*y* = 1 / (64.92 − 0.90*x*)) (GEE, random term: site)

In accordance with our SEM results, the analysis of mean wing asymmetry per cage showed a decrease with increasing wing centroid size values (Fig. 7: *χ*^2^ = 9.77, df = 1, *P* = 0.0018, *R*^2^ = 0.02; *y* = 1 / (16.89 + 3.77*x*)). Additionally, the analysis of mean wing asymmetry per cage showed a significant increase with increasing maximum temperatures (°C) (Fig. 8: *χ*^2^ = 38.60, df = 1, *P* < 0.0001, *R*^2^ = 0.15; *y* = 1 / (64.92 − 0.90*x*)).

## Discussion

We explored how microclimatic differences of the larval breeding habitat in a heterogeneous urbanised landscape affect juvenile survival and development time, as well as fitness-related parameters of emerging *Cx. pipiens* s.s./*Cx. torrentium* adults. Our results demonstrate that urbanisation index significantly affects the breeding habitat temperatures leading to significant changes in the analysed mosquito life-history traits.

The SEM revealed that increasing UI increased the mean temperature in the breeding sites, which shortens the developmental time of mosquitoes. Additionally, mean temperature has a direct effect on the wing centroid sizes of adults, which in turn had a direct effect on wing asymmetry. According to our SEM, mean temperature did not have a direct effect on the emergence rate of adults. This was likely because our model only tested for linear relationships, while adult emergence turned out to have a polynomial relationship with mean temperature.

In agreement with previous studies [20, 21], we found that urbanisation had an important influence on the microclimate of breeding sites across a heterogeneous urbanised area. The breeding sites in vegetation-dominated areas were up to 4 °C cooler than in urban areas with very high imperviousness. This microclimatic temperature difference distinctly influenced larval developmental time. A recent review confirms that *Cx. pipiens* developmental time decreases as temperature increases [73]. According to the so-called ‘temperature–size rule’, an increased growth rate due to higher environmental temperatures will also lead to a reduction in body size in most ectotherms [74–76]. This biological phenomenon is confirmed by our wing centroid size results, as there is a decrease in the wing centroid size of *Cx. pipiens* s.s./*Cx. torrentium* mosquitoes at higher mean temperatures.

Furthermore, the inverted U-shaped survival rate curve in this study agrees with previous observations on the thermal response for larval survival reviewed by Moser et al. [73], although there is a high variability in the findings of several authors in the same or closely related species. For instance, Loetti et al. [77] found the maximum survival of *Cx. pipiens* first-instar larvae to adulthood to be 76% at constant 25 °C and a survival range between 10 and 33 °C. Oda et al. [78] found an 80% survival for *Cx. quinquefasciatus* at a constant 25 °C, while survival was 95% at a constant 30 °C. In another study, field-collected *Cx. pipiens* juveniles from MD, USA, showed high survival probabilities (over 80%) when reared at constant temperatures between 16 and 32 °C [28]. However, these findings were mostly based on studies conducted under constant temperatures and using mosquito populations from laboratory strains rather than assessing the effect of naturally fluctuating field temperatures on larvae from wild populations. This high variability in experimental conditions and results underlines the importance of including specimens collected in the field and fluctuating temperatures in insect performance studies to accurately understand such temperature-dependent dynamics [79].

With our approach, we were interested in studying potential fitness differences among adults bred in different areas. For this, we tested the adult survival time under heat stress (constant 31 °C). Our results suggest that withstanding higher mean temperatures in their breeding sites as larvae did not enhance heat tolerance in adult mosquitoes, as adults bred in areas with warmer temperatures had a shorter survival time. This outcome appears to contradict the predictions of thermal acclimation theory, which states that exposure to sublethal thermal stress during early life stages can induce physiological mechanisms that improve tolerance in later stages [80]. Thermal acclimation from the juvenile to the adult stage has been previously demonstrated in *Cx. pipiens* by Gray [81]; however, before, adults were allowed to acclimate at different temperatures for several days before being tested for thermal tolerance. Overall, the survival time of adult mosquitoes under heat stress was relatively short (ranging from ~4 to ~6.5 days) compared with the results from other studies. Andreadis et al. [82] tested the adult longevity of a field-derived population of *Cx. pipiens* biotype pipiens at several temperatures and reported a decrease in adult lifespan at higher temperatures. However, even at the hottest temperature (30 °C) adults lived notably longer than in our study (males ~8 days, females ~11 days), possibly explained by a general better adaptation to warmer temperatures of the local mosquito populations in Greece. Spanoudis et al. [83] reports that *Cx. pipiens* biotype molestus adults reared at constant and fluctuating temperature of 32.5 °C had a lifespan ranging from 1.5 to 8 days after emergence. This once again supports the idea of high variability in life-history traits and susceptibility to environmental fluctuations among *Culex* populations, as pointed out by Ciota et al. [28]. Importantly, physiological adaptations to heat stress may involve trade-offs that compromise other fitness traits [84–86], potentially explaining the reduced adult longevity observed in our study. A recent study by Karpova et al. [87] showed that *Drosophila melanogaster* larvae subjected to heat stress exhibited decreased resistance and survival to acute heat stress as adults. This reinforces the idea that early life thermal stress can have lasting detrimental effects on adult fitness.

The wing size of *Cx. pipiens* s.s./*Cx. torrentium* mosquitoes significantly decreased at higher mean breeding habitat temperatures. This is in line with the above-mentioned ‘temperature–size rule’ [74–76] and agrees with other studies on *Culex* species [28, 77]. Additionally, wing asymmetry in *Cx. pipiens* s.s./*Cx. torrentium* mosquitoes decreased with higher wing centroid sizes, whereas it increased with higher maximum temperatures. This suggests that a faster development at higher temperatures leads to smaller organisms with higher asymmetries, possibly indicating environmental stress. In the past years, fluctuating asymmetry has been increasingly used as an indicator of different environmental stressors, although with very variable evidence among taxa, traits and stressors [37]. In line with our results, Mpho et al. [33] showed that thermal stress during larval development caused an increase in wing fluctuating asymmetry in adult *Cx. pipiens*. However, the extent to which fluctuating asymmetry translates into measurable changes in fitness is not fully understood. Further research is necessary to elucidate if and how wing asymmetry influences individual performance and population dynamics in mosquitoes.

It is worth discussing that despite our wing centroid size and wing asymmetry models being significant, the marginal *R*^2^, i.e. the proportion of variance explained by the fixed factor alone, and *R*^2^ values were very low. Our results had obvious clustering of data points. These clusters are neither a product of trial nor land use class, which led us to assume that they might be due to differences in wing centroid sizes within *Cx. pipiens* s.s./*Cx. torrentium.* This would explain the high variance in our data. The relationship between wing centroid size and wing asymmetry, as well as wing asymmetry and temperature stress, should be further analysed, including any possible consequences for individual fitness.

A valid point of criticism is that urbanised areas experienced quicker evaporation in breeding containers, leading to increased nutrient concentration as water levels fell below 0.8 L. While the methodology accounted for this by refilling the containers, it raises concerns about the consistency of nutrient levels. Nevertheless, the lack of observed differences in mean developmental time and survival probability among mosquitoes from different land use classes suggests that nutrient limitation or larval competition are unlikely to have impacted fitness. Carrieri et al. [88] report higher larval densities naturally occurring in artificial breeding sites detected in Italy (with the highest density being 110 larvae per litre); therefore, we assume that 30 larvae per litre is low density and expect minimal intraspecific competition. Furthermore, our results show that wing centroid size was significantly smaller in mosquitoes developing in breeding sites with higher mean temperatures. This pattern aligns with the ‘temperature–size rule’ [74–76], whereby elevated developmental temperatures accelerate larval growth but result in reduced adult size, possibly outweighing any positive effects of a higher nutrient concentration.

Our study demonstrated that variability in breeding site temperatures will produce adult mosquitoes with different body size and longevity [89–91]. However, how exactly these traits affect transmission cycles is still being discussed and might be highly vector-pathogen specific. For instance, Alto et al. [92] found evidence of size-dependent vector competence in *Ae. albopictus* and *Ae. aegypti* females, with smaller females being more likely to become infected and disseminate dengue virus. The opposite was found by Westbrook et al. [93] in a laboratory study in which *Ae. albopictus* females with larger bodies were more likely to be infected with CHIKV than smaller-bodied females reared at high temperatures. In their study, Brass et al. [30] examine the effects of phenotypic plasticity on disease dynamics by comparing the wing lengths of infected mosquitoes. They concluded that *Ae. albopictus* mosquitoes with wing lengths exceeding the population average have longer lifespans and can more likely survive the extrinsic incubation period of the virus. These above-mentioned studies, together with other works such as Juliano et al. [94] or Evans et al. [29], highlight the importance of integrating vector traits into transmission models to accurately assess the local risk of mosquito-borne disease outbreaks.

## Conclusions

The findings of this study highlight the significant influence of microclimatic variation across urbanised areas on the development and fitness traits of *Culex pipiens* s.s./*Cx. torrentium* mosquitoes. Urban sites, characterised by higher breeding site temperatures as compared with vegetation-dominated sites, led to faster larval development, smaller wing centroid sizes and decreased adult survival under heat stress. These results emphasise the need to incorporate fine-scale microclimatic data into predictive models to more accurately assess mosquito-borne disease risks in urban environments.

## Supplementary Information

Additional file 1 (Figure S1. Representative image of a Culex pipiens s.s. wing with the 18 landmarks used to carry out the geometric morphometric wing analysis and calculate the wing centroid size.)

## Data Availability

Data supporting the main conclusions of this study are included in the manuscript.

## Abbreviations

UI: Urbanisation index
IMD: Imperviousness density
TCD: Tree cover density
SEM: Structural equation modelling
