# Supplementary figures and images for: Carry-over effects in Culex species along a land use gradient with differences in microclimatic conditions
Source: Parasit Vectors. 2025 Jul 4;18:256. doi: 10.1186/s13071-025-06903-y (PMC12228285; doi:10.1186/s13071-025-06903-y)

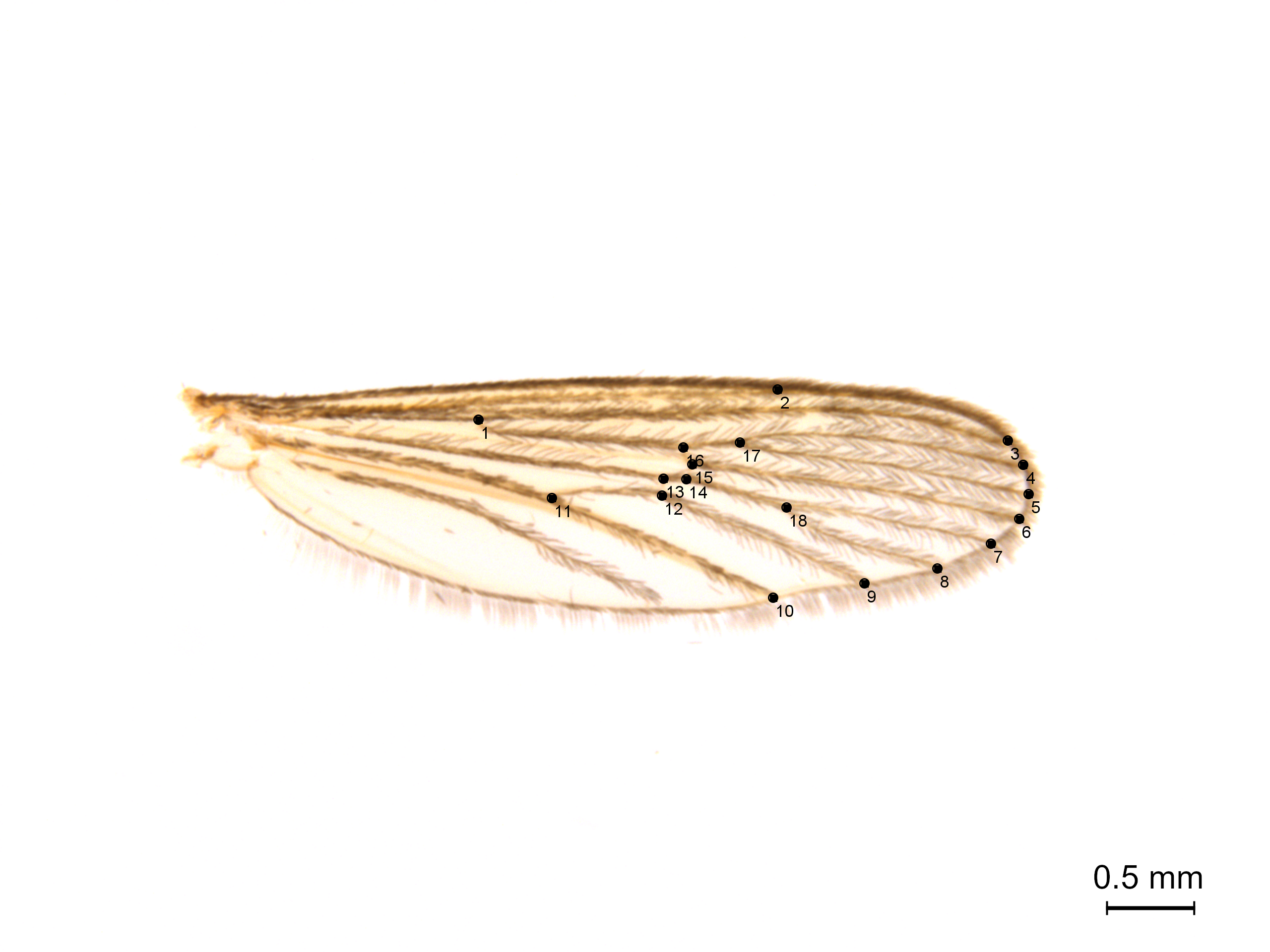

Supplement: Supplementary file 1 — Additional file 1 (Figure S1. Representative image of a Culex pipiens s.s. wing with the 18 landmarks used to carry out the geometric morphometric wing analysis and calculate the wing centroid size.) [file 13071_2025_6903_MOESM1_ESM.tiff]
